# Peripapillary vessel density in eyes with cone-rod dystrophy

**DOI:** 10.1371/journal.pone.0296167

**Published:** 2024-01-29

**Authors:** Masato Shinozuka, Mizuho Arai, Yumeno Hirayama, Yuna Uechi, Shohei Kawasaki, Kazuyoshi Okawa, Yume Iwashita, Misa Miyazato, Kazushi Hirono, Kentaro Nakamura, Tatsuya Inoue, Ryo Asaoka, Yasuo Yanagi, Maiko Maruyama-Inoue, Kazuaki Kadonosono

**Affiliations:** 1 Department of Ophthalmology and Micro-Technology, Yokohama City University, Kanagawa, Japan; 2 Department of Ophthalmology, Seirei Hamamatsu General Hospital, Shizuoka, Japan; 3 Seirei Christopher University, Shizuoka, Japan; St. Marianna University School of Medicine, JAPAN

## Abstract

**Purpose:**

To compared the vessel density (VD) around the optic nerve head (ONH) in eyes with cone-rod dystrophy (CORD) and healthy control eyes in a sector-wise manner and to investigate the relationship between VD around the ONH and visual function in CORD eyes.

**Methods:**

Twenty-six eyes in 14 CORD patients and 25 eyes in 25 healthy control subjects were examined. Using OCT angiography images, the VDs in the superficial and deep capillary plexus at the macula (sVDm and dVDm) and those around the ONH in the superior, temporal, inferior and nasal region (VDnh_s, VDnh_t, VDnh_i, and VDnh_n, respectively) were measured for each eye. Patient age, visual acuity (VA) and VDs were then compared between two groups. Moreover, the relationships between VA and the VDs were analyzed using a linear mixed model and AICc model selection.

**Results:**

No significant difference in age was seen between the CORD and control groups (*p* = 0.87, Wilcoxon rank sum test), but the VA was significantly lower in the CORD group (*p*<0.0001). Both sVDm and dVDm were significantly lower in the CORD eyes than in the control eyes (both *p*<0.0001). Among VDnh_s, VDnh_t, VDnh_i, and VDnh_n, however, only VDnh_t differed significantly between the CORD and control groups (*p* = 0.035). Among age, VDnh_t, dVDm, and sVDm, the optimal model for VA included only VDnh_t and dVDm.

**Conclusions:**

In addition to the VD in the deep capillary plexus at the macula, the measurement of temporal VD around the ONH might be useful for predicting visual function in eyes with CORD.

## Introduction

Cone-rod dystrophy (CORD) is an inherited retinal dystrophy caused by primary cone photoreceptor cell dysfunction [1, 2]. Several causative genes have been identified to date (https://sph.uth.edu/retnet/), and the genetic basis of CORD is highly heterogeneous [3]. In contrast to retinitis pigmentosa (RP), CORD is characterized by the impairment of cone photoreceptor cells and central vision loss, a decreased ellipsoid zone (EZ) intensity on optical coherence tomography (OCT) measurements, and abnormal autofluorescence in the affected area on fundus autofluorescence measurements (FAF). CORD patients present with decreased visual acuity (VA), impaired retinal sensitivity and color vision loss and sometimes demonstrate peripheral visual field loss at the end stage, followed by rod photoreceptor impairments [4]. Several studies have revealed a structure-function relationship in eyes with CORD, such as the correlation between VA and residual EZ and between retinal sensitivity and the abnormal autofluorescent area [5, 6].

OCT angiography (OCTA) is a relatively new, non-invasive imaging modality that quantitatively evaluates the perfusion status in different retinal layers [7]. Yilmaz et al. reported that a decreased vessel density was observed in both the superficial capillary plexus (SCP) and the deep capillary plexus (DCP) at the macula in eyes with cone dystrophy, compared with the control group [8]. Furthermore, VA was associated with vessel density in SCP and DCP, except for the foveal SCP, suggesting a significant correlation between visual function and vessel density in the macula in patients with cone dystrophy. In other inherited retinal diseases, such as RP, microvascular changes in the macula have been reported [9]. We also recently reported that the vessel density at the macula was closely related to VA in eyes with RP [10]. In addition, the relationship between the vessel density around the optic nerve head (ONH) was also associated with central visual field deterioration. On the other hand, the influence of sector-wise vessel density measurements around the ONH on visual function might differ across areas; for instance, temporal vessel density is closely associated with macular perfusion status, but this is not the case at other regions. Therefore, the present study aimed to compare the vessel density around the ONH in CORD eyes with that in healthy control eyes in a sector-wise manner to investigate the relationship between vessel density around the ONH and visual function in CORD eyes.

## Methods

The present study enrolled eyes with CORD and healthy control eyes. We included consecutive patients with CORD who visited our institute between December 1, 2020 and December 31, 2021. The diagnosis of CORD was based on a fundus examination, OCT measurements, FAF, and an electroretinogram (ERG). The exclusion criteria were eyes with (1) other ocular diseases, such as diabetic retinopathy, and (2) poor-quality OCTA images. This study was approved by the Ethics Committee of the Yokohama City University Medical Center (ID: F210900030). The study protocol adhered to the tenets of the Declaration of Helsinki, and written informed consent was obtained from all eligible patients.

The vessel density (VD) was measured using OCTA CIRRUS 6000 HD-OCT with AngioPlex (Carl Zeiss Meditec, Dublin, CA, USA) for each eye.

**Fig 1:**
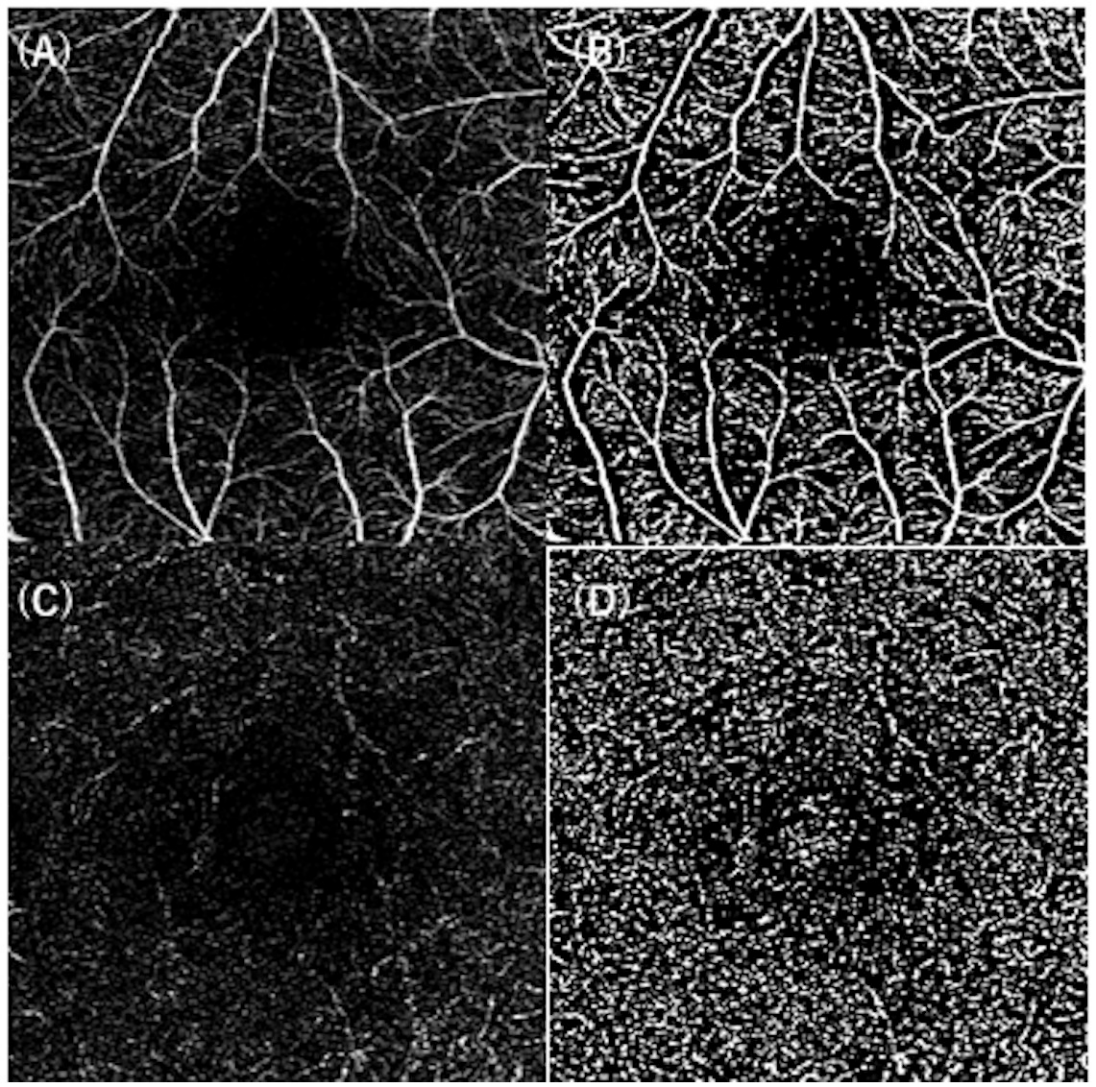
VD measurements at the macula in eyes with CORD. The OCTA image of the SCP (A) was binarized using the Otsu method (B). The DCP image (C) was also converted to a binarized image (D). The VDs were measured as sVDm and dVDm, respectively, for each eye. VD: vessel density, CORD: cone-rod dystrophy, SCP: superficial capillary plexus, DCP: deep capillary plexus.

The VD at the macular region was measured using 3 × 3-mm scans centered on the fovea. The OCTA images were imported into ImageJ software (ImageJ, V.2.0.0-rc-69/1.52i; NIH, Bethesda, Maryland, USA) and binarized using the Otsu method, and the VD was calculated in the SCP and the DCP as sVDm and dVDm, respectively (Fig 1).

**Fig 2:**
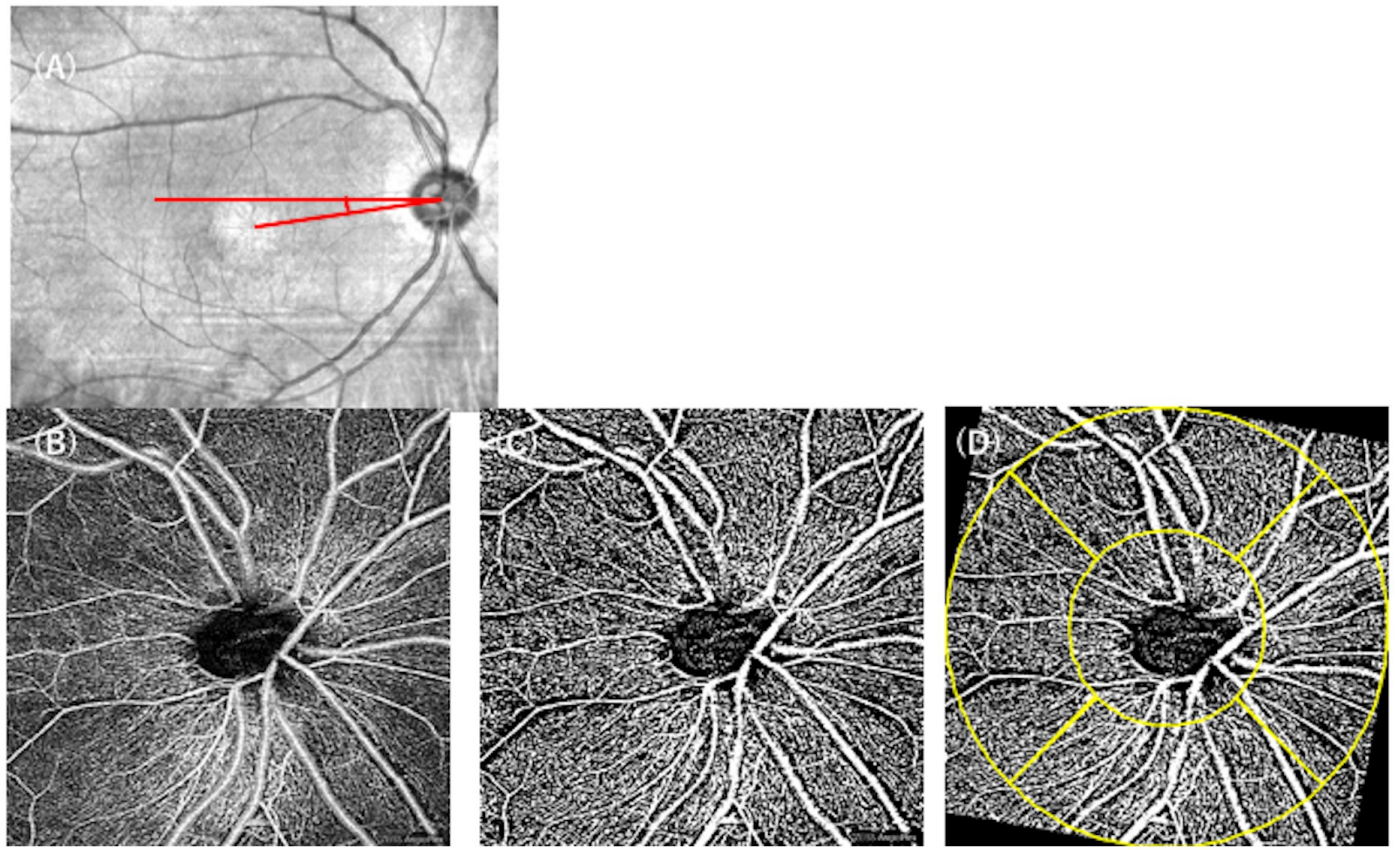
Measuring the VD around the ONH in eyes with CORD. Using the infrared image, the angle between the axis connecting the fovea and the optic disc center and the horizontal meridian was measured as the DFA (A, red). The OCTA image around the ONH (B) was binarized using the Otsu method (C). Then, according to the DFA, the binarized image was rotated, a ring-shaped grid (yellow) was superimposed, and the VD was calculated in each quadrant (D). VD: vessel density, ONH: optic nerve head, CORD: cone-rod dystrophy, DFA: disc-fovea angle.

Furthermore, 4.5 × 4.5-mm scans centered on the ONH were obtained for each eye. The ONH scans were automatically segmented into two layers: the whole retinal layer, and the radial peripapillary capillary (RPC) layer. The inner boundary of the whole layer was the internal limiting membrane (ILM), and its outer boundary was 70 μm beneath the inner plexiform layer (IPL). The RPC layer was segmented from the ILM to the posterior boundary of the retinal nerve fiber layer (RNFL). Using ImageJ software, the images were binarized using the Otsu method (Fig 2). Then, the vessel densities in the whole retinal layer (VDnh) and those in the RPC layer (sVDnh) were measured for each eye.

To calculate the disc-fovea angle (DFA), the infrared (IR) images were imported into ImageJ and the angle between the axis connecting the fovea and the optic disc center and the horizontal meridian was measured for each eye. Then, binarized OCTA images were rotated according to the DFA, respectively. To analyze the vessel density in a sector-wise manner, a ring-shaped grid centered on the ONH (inner circle, 1.5-mm diameter; outer circle, 4.5-mm diameter) was superimposed onto the OCTA images using ImageJ software (Fig 2D). Then, the vessel density in each quadrant (superior, temporal, inferior, nasal) was measured using the Otsu method, and these values were used for the statistical analysis (VDnh_s, VDnh_t, VDnh_i, VDnh_n, sVDnh_s, sVDnh_t, sVDnh_i, and sVDnh_n).

### Statistical analysis

The values of age, logMAR VA, and the VDs (sVDm, dVDm, VDnh_s, VDnh_t, VDnh_i, VDnh_n, sVDnh_s, sVDnh_t, sVDnh_i, and sVDnh_n) were compared between the CORD and control groups using the Wilcoxon rank sum test. A linear mixed model and a Tukey multiple comparison were conducted for VDnh_s, VDnh_t, VDnh_i and VDnh_n in the CORD and control groups, respectively. Moreover, the relationships between VA and age, VDnh_t, dVDm, and sVDm were investigated using the linear mixed model.

The linear mixed model is equivalent to an ordinary linear regression in that the model describes the relationship between the predictor variables and a single outcome variable. However, a standard linear regression analysis assumes that all observations are independent of each other. Using the linear mixed model, measurements were nested within subjects and, as a result, measurements were grouped within subjects to reduce the possible bias of including both eyes of one patient [11, 12]. Then, the model selection (round robin method; selection from all 2^4^ patterns) was performed to identify the optimal model for VA using the second-order Akaike information criterion (AICc) index, which provides an accurate estimation even when the sample size is small [13]. The selected variables in the optimal model were regarded as statistically significant. All statistical analyses were performed using the statistical programming language R (ver. 3.4.3, The R Foundation for Statistical Computing, Vienna, Austria).

## Results

The present study enrolled 14 CORD patients (7 female and 7 male) and 25 healthy control subjects (11 female and 14 male) (Tables 1 and 2). Out of 28 CORD eyes, two eyes were excluded from the study because of poor-quality OCTA images. The mean (± standard deviation, SD) age was 54.8 ± 16.2 years in the CORD group and 52.8 ± 13.2 years in the control group; this difference was not significant (*p* = 0.87, Wilcoxon rank sum test). The logMAR VA was significantly deteriorated in the CORD group, compared with that in the control group (0.455 ± 0.422 vs. -0.071 ± 0.045, *p* < 0.0001, Wilcoxon rank sum test).

**Table 1:** Demographic data.

	CORD	Control	*p* value
Number of eyes	26	25	
Age	54.8 ± 16.2	52.8 ± 13.2	0.87
LogMAR VA	0.455 ± 0.422	-0.071 ± 0.045	<0.0001
sVDm	28.9 ± 7.0	38.1 ± 3.9	<0.0001
dVDm	26.6 ± 6.8	35.4 ± 2.2	<0.0001
VDnh_s	49.7 ± 4.9	50.4 ± 3.9	0.83
VDnh_t	45.6 ± 4.3	48.5 ± 4.0	**0.035**
VDnh_i	47.9 ± 5.4	50.0 ± 4.2	0.17
VDnh_n	44.4 ± 4.0	45.0 ± 4.8	0.89
DFA	8.39 ± 5.4	7.76 ± 4.0	0.86

CORD: cone-rod dystrophy, logMAR VA: logarithm of the minimum angle of resolution visual acuity, sVDm: vessel density in the retinal superficial layer at the macula, dVDm: vessel density in the retinal deep layer at the macula, VDnh_s: vessel density around the optic nerve head in the superior region, VDnh_t: vessel density around the optic nerve head in the temporal region, VDnh_i: vessel density around the optic nerve head in the inferior region, VDnh_n: vessel density around the optic nerve head in the nasal region, DFA: disc-foveal angle

**Table 2:** Clinical aspects of the study subjects.

Case	Age	Gender	Decimal VA (R/L)	FAF pattern (R/L)	photopic ERG a-wave amplitude (μV) (R/L)	photopic ERG b-wave amplitude (μV) (R/L)
1	73	F	0.3/0.2	patchy/patchy	21.8/22.3	53.5/39.8
2	73	F	0.8/0.01	patchy/patchy	21.8/6.5	67.5/39.3
3	73	M	0.09/0.09	hypofluorescent/hypofluorescent	39.0/25.3	31.5/28.3
4	67	M	0.5/0.15	patchy/patchy	13.3/13.5	50.5/54.8
5	45	M	0.2/0.15	hypofluorescent/hypofluorescent	40.8/32.8	56.0/58.5
6	52	M	1.0/0.4	patchy/patchy	24.0/20.3	54.8/35.8
7	75	M	0.02/0.3	hypofluorescent/patchy	20.3/34.5	51.3/48.5
8	37	F	1.2/1.0	patchy/patchy	13.5/4.5	29.8/25.5
9	40	F	0.08/0.1	hypofluorescent/patchy	1.0/0	19.5/20.0
10	43	M	0.1/0.15	patchy/patchy	38.0/37.0	63.8/76.3
11	48	F	0.5/0.2	patchy/patchy	25.5/34.5	32.8/41.0
12	81	F	1.2/0.8	patchy/patchy	13.3/20.5	51.3/51.8
13	49	M	0.8/0.9	patchy/patchy	15.8/21.5	14.3/21.5
14	31	F	1.2/1.2	patchy/patchy	30.5/20.0	82.5/64.0

VA: visual acuity, FAF: fundus autofluorescence, ERG: electroretinogram

Both the sVDm and the dVDm were significantly reduced in the CORD eyes, compared with the control group (both *p* < 0.0001, Wilcoxon rank sum test). On the other hand, among VDnh_s, VDnh_t, VDnh_i, VDnh_n, sVDnh_s, sVDnh_t, sVDnh_i and sVDnh_n, the only parameter with a significant difference between the CORD and control groups was VDnh_t (*p* = 0.035, Wilcoxon rank sum test).

**Fig 3:**
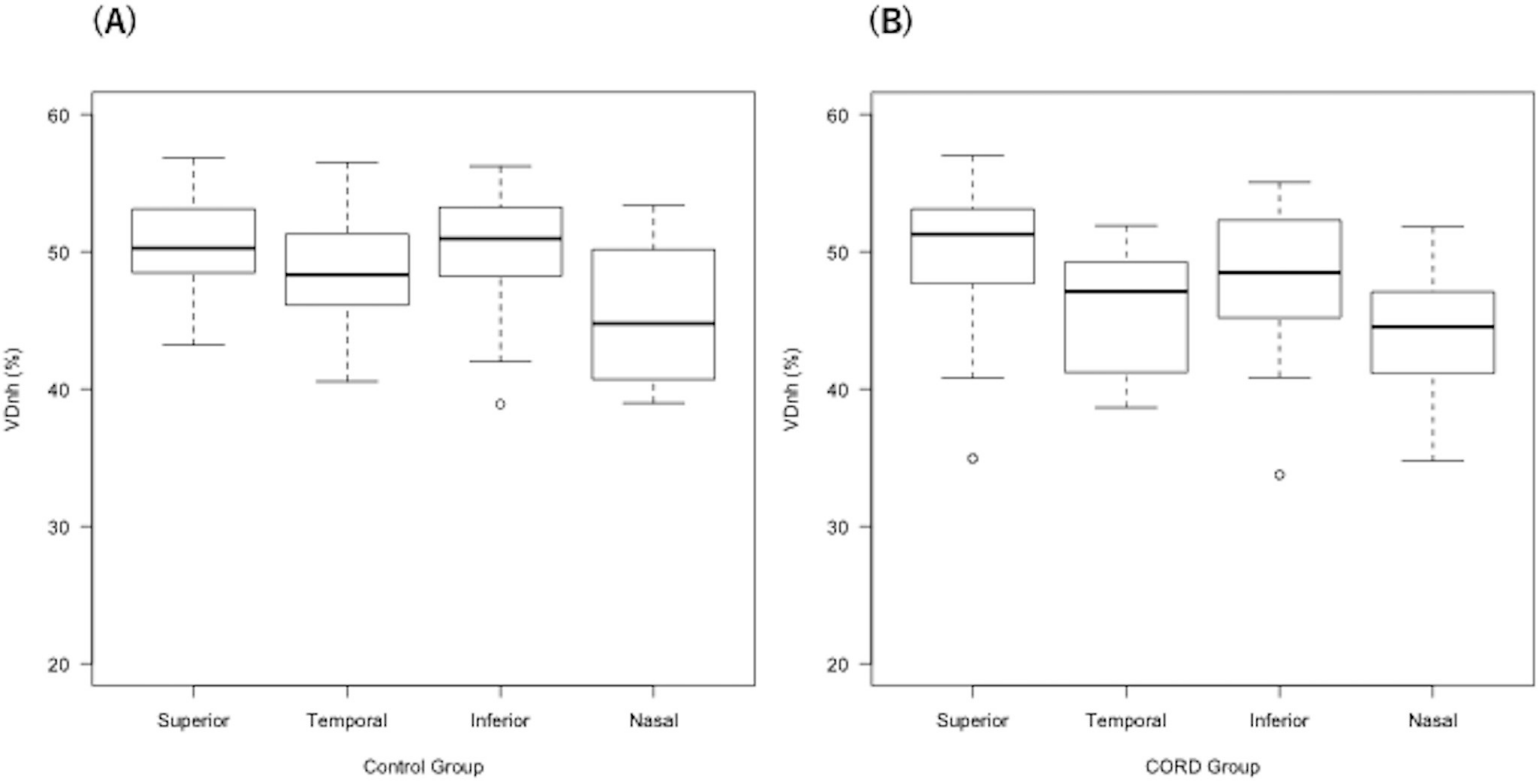
Sector-wise VD measurements around the ONH in the CORD and control groups. The nasal VD was significantly lower in the control group (A), while the temporal and nasal VDs were lower than the superior and inferior VDs in the CORD group (B, linear mixed model and Tukey multiple comparison test). VD: vessel density, ONH: optic nerve head, CORD: cone-rod dystrophy.

The linear mixed model and Tukey multiple comparison test suggested that VDnh_n was significantly lower than VDnh_s, VDnh_t and VDnh_i in the control group (*p* < 0.001, *p* = 0.022, *p* < 0.001, respectively, Fig 3A). On the other hand, in the CORD group, VDnh_n was significantly lower than VDnh_s and VDnh_i (both *p* < 0.001), but a significant difference was not seen between VDnh_n and VDnh_t (*p* = 0.48, Tukey multiple comparison test). Moreover, VDnh_t was significantly lower than VDnh_s and VDnh_i in the CORD group (*p* < 0.001, *p* = 0.046, respectively; Fig 3B).

A multivariate analysis followed by AICc model selection suggested that among age, VDnh_t, dVDm, and sVDm, the optimal model for logMAR VA included only VDnh_t and dVDm, as follows.


$$LogMAR\;VA=1.78–0.0066\;(\pm 0.0042)\times VDnh\_t–0.010\;(\pm 0.0029)\times dVDm\left(AICc=24.2\right)$$

## Discussion

In the present study, the VD around the ONH measured using OCTA was compared between CORD and healthy control eyes in a sector-wise manner. The temporal VD around the ONH was reduced in CORD eyes, in addition to reductions in the SCP and DCP at the macula. Furthermore, the temporal VD around the ONH and the VD in the deep retinal layer at the macula were significantly associated with VA.

The topic of the current research is clinically relevant because OCTA has been shown to provide new insights into structure-vasculature-function relationships in other ophthalmic pathologies, such as glaucoma [14]. Whether microvascular changes are directly involved in the pathogenesis of CORD remains unclear, but it may be useful to monitor the vessel density in CORD to predict the progression of visual impairment in the clinical settings. Our current results suggested the association between the vessel density and visual acuity. As cone photoreceptor cell impairment in CORD is caused by genetic mutation, it is highly possible that cone photoreceptor cell loss precedes the VD reduction, followed by visual impairment, although, there is also a possibility that the decrease in oxygen consumption influences visual function to some extent. Consistent with previous work by Yilmaz et al. [8], our current study suggested that the VDs in both the SCP and the DCP at the macula (sVDm and dVDm) were reduced, compared with the values in healthy control subjects. The reduction in the VD in the SCP might result from the inner retinal layer being affected by outer retinal degeneration. On the other hand, Sabbaghi et al. suggested that the VDs in the parafoveal and perifoveal DCP were reduced in CORD eyes [15]. In the present study, a multivariate analysis suggested that dVDm, but not sVDm, be included in the optimal model for VA. The VD of DCP at the macula may reflect visual function more sensitively in eyes with CORD.

Our comparative study demonstrated that the temporal VD around the ONH (VDnh_t) was deteriorated in CORD eyes, whereas the VDs in other peripapillary regions (VDnh_s, VDnh_i, VDnh_n) were not reduced. We previously reported that RP patients demonstrated a reduced vessel density around the ONH in the whole retinal layer, but not in the superficial layer [10]. Similarly, the current results suggested that the temporal VD in the whole layer, but not in the RPC layer, was deteriorated, in CORD eyes. Given that photoreceptor cell death primarily occurs in both RP and CORD, the finding that OCTA changes were less prominent in the RPC layer than in the whole retinal layer seems reasonable. Taken together, our findings suggest that OCTA measurements might be useful for the diagnosis of CORD. Furthermore, since the optimal model for logMAR VA included VDnh_t and dVDm, sector-wise VD measurements around the ONH, especially in the temporal region, might be important for predicting visual function in eyes with CORD.

The limitations of the present study include its retrospective nature and a relatively small sample size. Additionally, the automated segmentation was difficult to perform for some CORD eyes, and the VD measurements might be unreliable. Finally, visual field is an important visual function in clinical settings. All the eyes with CORD had undergone simultaneous visual field measurements using a Humphrey field analyzer (HFA; Carl Zeiss Meditec, Dublin, CA) in the present study. However, eight of 26 enrolled CORD eyes were considered to have unreliable results because eyes with (i) fixation losses ≥20%, (ii) false-positive rates ≥15%, or (iii) false-negative rates ≥33% were excluded, as determined by SITA-Standard [16]. The microperimeter is a fundus-controlled perimetry and is useful for assessing central visual field in macular diseases. It would be interesting to analyze the association of VDs with the visual field measured using microperimetry in CORD eyes.

In conclusion, the temporal VD around the ONH was reduced in CORD eyes and was related to VA, in addition to the VD in the deep retinal layers at the macula, suggesting the usefulness of measuring the VD around the ONH for accurately predicting visual function in eyes with CORD.

## Supporting information

S1 Data (CSV)
